# Worm in the Water: Diagnosing Urinary Ascariasis in an Elderly Patient Through Routine Microscopy

**DOI:** 10.7759/cureus.89317

**Published:** 2025-08-04

**Authors:** Rayana Shrestha, Nava R Sharma, Abhishesh Wagle, Madalasa Pokhrel

**Affiliations:** 1 Department of Internal Medicine, Institute of Medicine, Maharajgunj, Kathmandu, NPL; 2 Department of Internal Medicine, Maimonides Medical Center, Brooklyn, USA; 3 Department of Medicine, Manipal College of Medical Sciences, Pokhara, NPL; 4 Department of Infectious Diseases, Maimonides Medical Center, Brooklyn, USA

**Keywords:** ascaris lumbricoides, ectopic parasitic infection, suburban healthcare nepal, urinary ascariasis, urine microscopy

## Abstract

Ascariasis is primarily an intestinal parasitic infection, with ectopic migration to the genitourinary tract, representing an uncommon clinical entity. Such presentations are infrequently reported and may pose a diagnostic challenge, particularly in the absence of gastrointestinal symptoms.

We report a case of an 88-year-old female from a suburban region of Nepal, who presented with bilateral iliac pain and dysuria of three days’ duration. Physical examination was notable for mild lower abdominal tenderness, with stable hemodynamic parameters. Laboratory investigations revealed leukocytosis with neutrophilic predominance. Urine microscopy demonstrated the presence of a motile helminth, subsequently identified as *Ascaris lumbricoides*. The patient denied gastrointestinal complaints, fever, or any prior history suggestive of helminthic infection.

This case illustrates a rare presentation of urinary ascariasis diagnosed through conventional urine microscopy in the absence of gastrointestinal manifestations. The diagnosis was established using basic diagnostic modalities available in a suburban healthcare setting, underscoring the continued relevance of routine microscopy in detecting atypical parasitic infections. Although rare, urinary ascariasis should remain a differential consideration in patients from endemic areas presenting with urinary tract symptoms. In resource-limited settings, fundamental laboratory techniques continue to play a critical role in identifying uncommon infectious etiologies.

## Introduction

Ascariasis, caused by the nematode *Ascaris lumbricoides*, affects approximately 0.8-1.2 billion people globally, making it the most prevalent helminthic infection worldwide [1]. Despite ongoing efforts involving improved sanitation and mass drug administration programs, recent systematic reviews estimate that over 700 million individuals remain infected [2]. The disease is particularly endemic in tropical and subtropical regions, including South Asia, where Nepal continues to face a significant burden of soil-transmitted helminths [1].

The typical lifecycle of *A. lumbricoides* involves ingestion of embryonated eggs, followed by larval migration through the lungs and eventual maturation in the small intestine [3]. While most infections are asymptomatic or limited to gastrointestinal manifestations, ectopic migration to atypical anatomical sites may occur and lead to clinically significant complications [3]. Among these, urinary tract involvement is extremely rare, with fewer than 50 cases reported in the literature over the past several decades [4].

In Nepal, rural and suburban areas remain vulnerable to ongoing transmission of helminths, as demonstrated by studies in regions such as Chitwan [5]. Diagnosis of parasitic infections in resource-limited settings often depends on basic laboratory investigations, which remain indispensable in identifying atypical presentations.

This case report describes a rare instance of urinary ascariasis diagnosed via routine urine microscopy in an elderly female in a suburban healthcare setting, underscoring both the unusual nature of the presentation and the diagnostic value of fundamental laboratory techniques in endemic, low-resource environments.

## Case presentation

An 88-year-old female from a suburban region in western Nepal presented to the emergency department with a three-day history of bilateral iliac pain and dysuria. The abdominal pain was described as dull, constant, and non-radiating, localized to the lower quadrants. Dysuria was present without accompanying urgency, frequency, hematuria, or nocturia. The patient denied any fever, chills, nausea, vomiting, diarrhea, or other gastrointestinal complaints.

Her past medical history was unremarkable. She had no known chronic illnesses, prior hospitalizations, or history of deworming. There was no recent travel outside her locality, and her family history and lifestyle factors were non-contributory. On examination, the patient was alert and oriented. Vital signs were stable: blood pressure was 100/80 mmHg, heart rate was 92 beats per minute, respiratory rate was 18 breaths per minute, and oral temperature was 98.6°F (37°C). Cardiovascular and respiratory examinations were within normal limits. Abdominal examination revealed mild tenderness in the bilateral lower quadrants without guarding, rebound, or palpable masses. Bowel sounds were normal. There was no costovertebral angle tenderness, and the genitourinary examination was unremarkable.

Initial laboratory investigations are summarized in Tables 1, 2. Notable findings included leukocytosis with neutrophilia, while eosinophil count remained within the normal range. Urinalysis showed a turbid appearance, acidic pH, and proteinuria. C-reactive protein and renal function tests were within normal limits. A stool sample could not be obtained during the initial visit due to the patient’s inability to provide a specimen.

**Table 1:** Blood investigations.

Test	Result	Reference range
White blood cell count	14,040 cells/µL	4,000–11,000 cells/µL
Neutrophils	89%	40–70%
Eosinophils	1%	1–4%
Hemoglobin	11.2 g/dL	12–16 g/dL (female)
Platelet count	Normal	150,000–400,000/µL
C-reactive protein	Normal	<5 mg/L
Serum creatinine	Normal	0.5–1.1 mg/dL (female)
Blood urea nitrogen (BUN)	Normal	7–20 mg/dL

**Table 2:** Urinalysis. HPF: high-power field.

Test	Result	Reference range
Urine pH	Acidic	4.5–8.0
Urine albumin	+2	Negative
Red blood cells (RBCs), HPF	2–4	0–2
Epithelial cells, HPF	6–8	0–5
White blood cells (WBCs), HPF	Occasional	0–5

**Figure 1:**
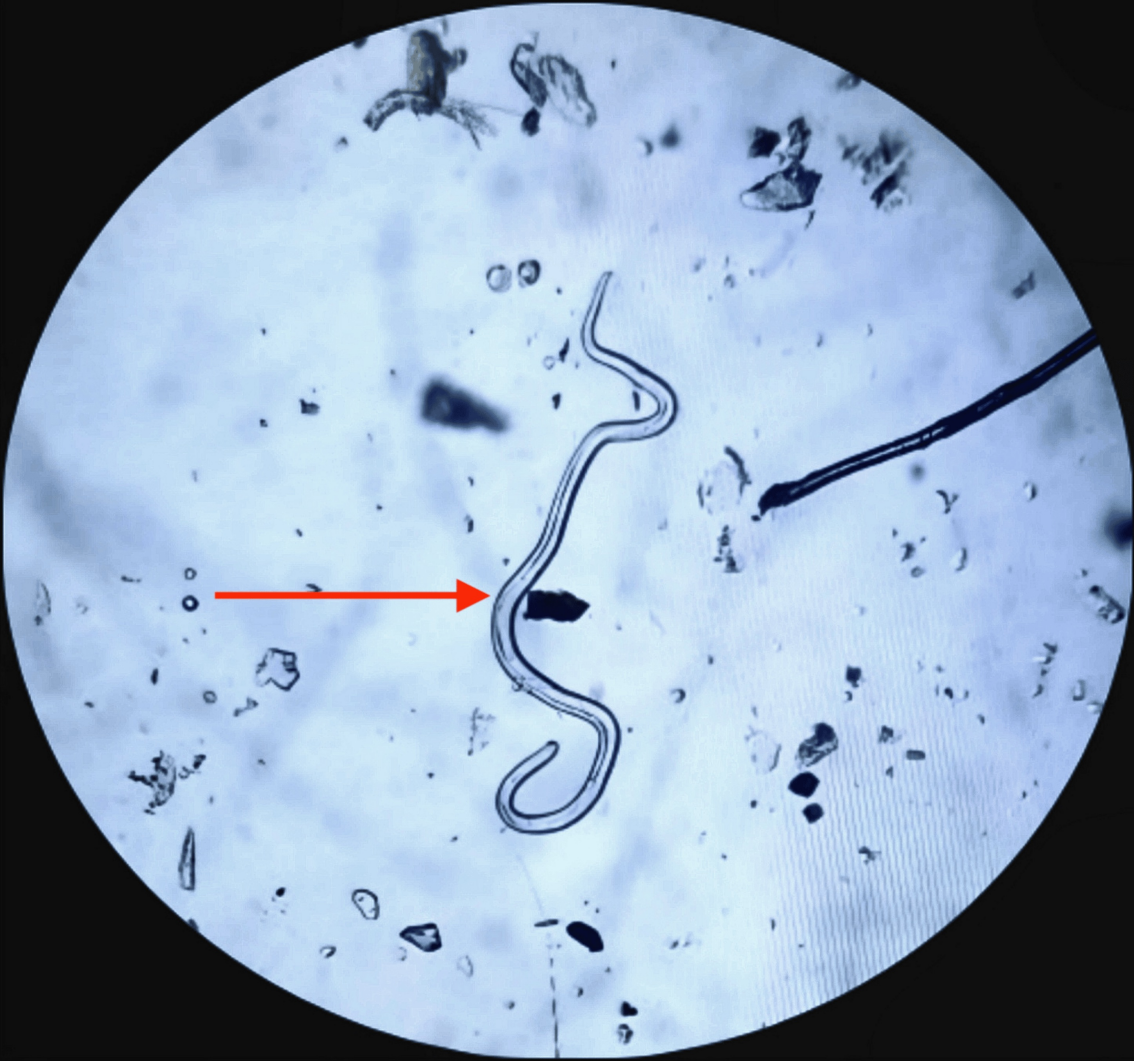
Urine microscopic image showing a parasitic worm indicated by the red arrow.

Microscopic examination of fresh urine revealed 2-4 red blood cells per high-power field, 6-8 epithelial cells per high-power field, occasional white blood cells, and most remarkably, a motile helminth consistent with *Ascaris lumbricoides*, measuring approximately 2-3 cm in length with cylindrical morphology and pointed ends, as shown in urine microscopy (Figure 1).

The patient was prescribed albendazole 400 mg as a single dose and counseled regarding the diagnosis. She was advised to return with a stool specimen and follow up in one week. At follow-up, she reported complete resolution of symptoms. Repeat urinalysis showed no evidence of parasites. She was educated on sanitation and personal hygiene to prevent future parasitic infections.

## Discussion

This case highlights a rare presentation of urinary ascariasis in an elderly female, diagnosed solely through routine urine microscopy in a suburban healthcare setting. The absence of gastrointestinal symptoms and the nonspecific nature of urinary complaints underscore the diagnostic challenge posed by such ectopic infections. Although *Ascaris lumbricoides* affects nearly one billion people worldwide and contributes substantially to disease burden in tropical and subtropical regions, urinary tract involvement remains exceptionally rare, with fewer than 50 cases reported in the literature [4-8].

The exact mechanism by which *Ascaris* reaches the urinary tract is not well understood. Proposed theories include migration through an enterovesical or rectovesical fistula, retrograde movement through the urethra, which is more plausible in females due to shorter urethral length, and abnormal larval migration during the pulmonary phase of the parasite’s lifecycle [4-9]. In this case, the absence of intestinal symptoms and the discovery of an adult worm in the urine suggest retrograde urethral migration as the most likely pathway.

Clinically, urinary ascariasis may present with striking features such as passage of worms in the urine, hematuria, urinary retention, or recurrent urinary tract infections [4,10]. Some reports describe patients observing worms emerging from the urethra during urination. In contrast, our patient experienced only mild lower abdominal discomfort and dysuria without hematuria or gastrointestinal involvement. This subtle presentation can easily lead to misdiagnosis, especially in older adults, where more common urological conditions may be considered first. An interesting finding in this case was the absence of peripheral eosinophilia, which is typically associated with helminthic infections. However, eosinophilia is more commonly observed during larval migration rather than with established adult worm infections localized in one area [11]. This case illustrates the continued relevance of direct parasitological methods, as the diagnosis was confirmed through urine microscopy.

The healthcare setting where this case was identified represents a suburban context in Nepal, where access to advanced diagnostic imaging or molecular tools may be limited. Nevertheless, a definitive diagnosis was achieved using simple and cost-effective urine microscopy. In communities where soil-transmitted helminths remain prevalent due to poor sanitation, such diagnostic methods remain essential [5,12]. This case is notable for several reasons. First, the patient’s advanced age is unusual, as most cases of urinary ascariasis are documented in children or young adults [13]. Second, the lack of gastrointestinal symptoms distinguishes this case from others where urinary involvement occurs alongside intestinal infection. Third, the diagnosis was made without the need for high-level diagnostics, reaffirming the diagnostic value of basic microscopy. Lastly, this case adds a rare geographic instance from Nepal, where reports of ectopic helminthiasis remain scarce.

Management followed standard treatment for ascariasis. A single 400 mg dose of albendazole was administered, and the patient responded well [6,14]. However, there were limitations. Stool analysis was not performed, so concurrent intestinal infection could not be assessed. Additionally, no imaging was conducted to evaluate possible anatomical routes of migration. Future studies should aim to establish clearer diagnostic pathways and treatment guidelines for urinary ascariasis, given its uncommon and varied presentations.

In summary, this case underscores the importance of considering parasitic infections in the differential diagnosis of nonspecific urinary symptoms in endemic regions. It also highlights the enduring utility of basic laboratory techniques such as urine microscopy in detecting rare parasitic infections, particularly in settings where advanced diagnostics are not readily accessible.

## Conclusions

Urinary ascariasis is a rare manifestation of a common parasitic infection that may present with nonspecific urinary symptoms and absence of typical gastrointestinal signs. In this case, diagnosis was made based on direct visualization of the worm in urine microscopy combined with compatible clinical features and response to treatment. However, definitive confirmation of causation is limited by the absence of advanced diagnostic procedures such as cystoscopy or imaging. This report highlights the utility of simple urine microscopy in resource-limited, remote settings for detecting unusual presentations of parasitic infections. Healthcare providers in endemic areas should maintain a high index of suspicion for ectopic parasitic infections, even with subtle urinary symptoms. Continued emphasis on sanitation, hygiene education, and maintaining basic diagnostic skills remains essential for managing such uncommon cases.
